# The Outcome of Hydroxychloroquine in Patients Treated for COVID-19: Systematic Review and Meta-Analysis

**DOI:** 10.1155/2020/4312519

**Published:** 2020-10-13

**Authors:** Teshale Ayele Mega, Temesgen Mulugeta Feyissa, Dula Dessalegn Bosho, Kabaye Kumela Goro, Getandale Zeleke Negera

**Affiliations:** ^1^Deaprtment of Pharmacology and Clinical Pharmacy, School of Pharmacy, College of Health Sciences, Addis Ababa University, Addis Ababa, Ethiopia; ^2^Department of Clinical Pharmacy, School of Pharmacy, Institute of Health, Jimma University, Jimma, Ethiopia

## Abstract

**Background:**

The pandemic of coronavirus disease 2019 (COVID‐19) caused by severe acute respiratory syndrome coronavirus 2 (SARS-CoV-2) resulted in an unprecedented public health challenge worldwide. Despite urgent and extensive global efforts, the existing evidence is inconclusive regarding the medications used for the treatment of COVID-19.

**Purpose:**

To generate an up-to-date evidence for the clinical safety and efficacy of hydroxychloroquine (HCQ) with or without azithromycin (AZ) among patients treated for COVID-19. *Data Source*. PubMed, Cochrane CENTRAL, LITCOVID, Web of Science, SCOPUS, BioRxiv, Embase, MedRxiv, and Wiley online library were searched from 2019/12/30 to 2020/05/23. *Study Selection*. Three investigators assessed the quality of the studies. *Data Extraction*. Data about study characteristics, effect estimates, and the quality of the studies were extracted by two independent reviewers and cross-checked by the third reviewer. *Data Synthesis*. The data of 6,782 (HCQ group, 3623; HCQ + AZ group, 1,020; control group, 2139) participants were included. HCQ was compared with standard care for virologic efficacy, disease progression, mortality, and adverse effects. HCQ was also compared with HCQ + AZ for QTc prolongation, admission to the intensive care unit, and mortality. The study found HCQ did not alter the rate of virologic cure (OR = 0.78; 95% CI: 0.39–1.56) and the risk of mortality (OR = 1.26; 95% CI: 0.66–2.39). The pooled prevalence for mortality was 5.8% (95% CI: 0.9%–10.8%). Moreover, HCQ did not impact disease progression (OR = 0.9; 95% CI: 0.36–2.29) but resulted in a higher risk of adverse effects (OR = 2.35; 95% CI: 1.15–4.8). HCQ was also compared against HCQ + AZ, and no difference was observed in QTc prolongation above 500 ms (OR = 1.11; 95% CI: 0.54–2.28), admission to the intensive care unit (OR = 0.92; 95% CI: 0.52–1.63), and mortality (OR = 0.88; 95% CI: 0.55–1.43). However, in the analysis of single-arm studies, about 11.2% (95% CI: 7.0%–15.5%) of patients have developed an absolute increase of QTc greater than 500 ms, and 4.1% (95% CI: 1.1%–7.1%) of patients discontinued their medication.

**Conclusion:**

This meta-analysis and systematic review, which included a limited number of poorly designed studies of patients with COVID-19, revealed HCQ is intolerable, unsafe, and not efficacious. Similarly, HCQ + AZ combination was not different from HCQ alone in curbing mortality and ICU admission.

## 1. Introduction

The pandemic of coronavirus disease 2019 (COVID‐19) caused by severe acute respiratory syndrome coronavirus 2 (SARS-CoV-2) resulted in an unprecedented public health challenge worldwide [1]. As of May 26, 2020, there were more than 5.3 million documented cases, and over 300,000 patients have succumbed to this disease globally [2]. The morbidity and mortality due to COVID-19 have found to increase with age and the presence of comorbid conditions such as diabetes, hypertension, coronary heart disease, or chronic obstructive lung disease [3].

With the rising death toll and a vaccine unlikely very soon, extensive global efforts are underway to develop safe and effective therapeutics against COVID‐19 [4]. Among the efforts undergoing to treat the disease, repurposing of old medications is a compelling strategy for which their safety profile, pharmacokinetics, and potential drug interactions are well studied [5].

Initially, a combination of lopinavir and ritonavir was utilized as the first‐line agent in Wuhan, China, the epicenter of the disease. However, a previous study [5] failed to show the beneficial clinical effects of this combination. Indeed, it has received considerable criticism from the scientific community [6].

Meanwhile, the aminoquinolines, chloroquine (CQ), and hydroxychloroquine (HCQ) have emerged as a potent inhibitor of SARS-CoV-2 in vitro, and some studies also demonstrated their clinical benefit among hospitalized COVID-19 patients [7–9]. On March 30, 2020, the United States Food and Drug Administration (FDA) granted emergency authorization that allowed the use of these drugs in hospitalized COVID-19-pneumonia [10].

To date, regardless of limited evidence, HCQ with or without azithromycin (AZ) is widely utilized in clinical settings to treat thousands of COVID-19 patients around the world [4]. The studies supporting the use of HCQ had suffered from methodological flaws including small sample size and ill quality of design creating difficulty in measuring the true clinical effects. The first study from France showed HCQ and AZ combination as an effective therapy for COVID-19. In this open-label non-randomized clinical trial, a total of 20 patients were treated with HCQ at a dose of 200 mg three times daily for 10 days, and the data showed a significant reduction in viral carriage at day 6 post-inclusion compared to controls (70.0% clearance by day 6 vs. 12.5% clearance by day 6 in control groups). Interestingly, the addition of AZ to HCQ (*n* = 6) resulted in a 100% virological cure on day 6 postinclusion, compared with 57.1% virological cure in the HCQ alone arm (*n* = 14) and 12.5% virological cure in the control arm (*n* = 16) [8]. Similarly, Million et al. [11] showed HCQ/AZ combination to be safe with a lower death rate. On the contrary, a more recent study conducted by Molina and his colleagues [12] failed to show evidence of a strong antiviral activity or clinical benefit of HCQ in combination with AZ for the treatment of hospitalized patients with severe COVID-19. Notably, the patients included in this study belonged to the severe COVID-19 category and had significant comorbidities including solid and hematological cancers, HIV, and obesity.

A large observational study conducted in the USA reported that HCQ use among patients hospitalized with COVID-19 was not associated with either a greatly lowered or increased risk of intubation or death [4]. A very recent study by Mahévas et al. [13] and Rosenberg et al. [14] also did not show significant differences in terms of in-hospital mortality among patients receiving HCQ with/without AZ compared with standard care. Besides, a systematic review by Sarma and his colleagues [15] concluded that treatment with HCQ had benefits in terms of fewer cases showing radiological progression, time to body temperature normalization, and the number of cough days compared to standard treatment. However, no difference was seen in terms of virological cure, death, or clinical worsening of the disease.

The safety of HCQ with/without AZ in COVID-19 patients, including cardiac arrest and QTc prolongation, was also investigated by several studies [14, 16]. Interestingly, both drugs can potentially cause QTc prolongation, leading to life-threatening ventricular arrhythmias and torsade de pointes [17]. Critically ill admitted COVID-19 patients with multiorgan failure and metabolic derangements and those having other drugs that can increase the risk of QTc prolongation are at greater risk [18]. A study conducted in New York recorded higher rates of cardiac arrest among patients receiving a combination of HCQ and AZ [14]. Similarly, Ramireddy et al. [19] reported a significant number of patients with QTc-interval prolongation, and the highest QTc values were recorded in those treated with a combination of HCQ and AZ.

Despite numerous studies with small sample size, the efficacy and safety of HCQ in COVID-19 patients remained unclear. Given the inconclusiveness of the existing evidence and awaiting findings from large randomized controlled clinical trials to clear the controversy, we conducted a systematic review and meta-analysis in the interim to investigate the safety and effectiveness of HCQ in the clinical setup.

## 2. Objective

The objective of this review is to synthesize an evidence for the safety, efficacy, and tolerability of HCQ with or without AZ among patients treated for COVID-19.

## 3. Methods

This review was described by the Preferred Reporting Items for Systematic Reviews and Meta-Analysis (PRISMA) framework. The studies were identified from PubMed, Cochrane CENTRAL, LITCOVID, Web of Science, SCOPUS, BioRxiv, Embase, MedRxiv, and Wiley online library. The search was conducted to include human studies published in English language from 2019/12/30 to 2020/05/23. The search terms included 2019‐nCoV, 2019 novel coronavirus, COVID‐19, coronavirus disease-2019, hydroxychloroquine, Plaquenil, and hydroxychloroquine sulphate. Details of the search strategy for some databases are annexed (Appendix A).

Study designs with single‐group prospective/retrospective observational studies and controlled clinical trials were pooled using meta-proportion, while prospective/retrospective observational studies and controlled clinical trials comparing HCQ with or without AZ versus usual care or HCQ with HCQ plus AZ were pooled using RevMan version 5.3. Controlled clinical trials with serious risk of bias were not included in the pooled analysis, and their findings were narrated descriptively.

## 4. Selection of Studies

After removing all irrelevant articles, TAM, TMF, and GZN independently reviewed articles for data quality and methodological validity using standardized critical appraisal instruments obtained from https://www.joannabriggs.org/assets/docs/jbc/...sr.../jbi-sr-protocol-template.docx. Any disagreement was handled by consulting KKG and DDB. Data extraction was handled by DD and KK using the standardized data extraction tool available at https://www.joannabriggs.org/assets/docs/jbc/...sr.../jbi-sr-protocol-template.docx.

### 4.1. Inclusion and Exclusion Criteria

Studies were considered if they included patients who received HCQ alone or in combination with other specific treatment modalities for COVID-19 infection. Both controlled clinical trials (CCTs) and observational studies with and without the comparator group were considered for inclusion. Data on at least one of the following outcomes had to be available for inclusion: virologic efficacy, mortality, disease progression, adverse effects, QTc prolongation, and drug discontinuation due to adverse effects (tolerability). Studies conducted among pediatric COVID-19 patients, case reports, preclinical studies, and studies that did not report outcomes with HCQ in COVID-19 were excluded.

### 4.2. Risk of Bias

The risk of bias for comparative clinical trials [8, 20–22] was assessed using the Cochrane risk of bias tool for randomized controlled studies [23]. The study by Gautret et al. [8] was a non-randomized clinical trial and hence assessed using the ROBINS‐I scale [24]. The results of the risk of bias for the studies included in the meta-analysis are found in Appendix B. The Modified Newcastle-Ottawa Quality Assessment scale [25] was used for observational studies, and the full results are presented in Appendix B.

### 4.3. Outcome Assessment and Statistical Analysis

The virologic efficacy, mortality, disease progression, adverse effects, QTc prolongation, and drug discontinuation due to adverse effects (tolerability) were assessed. Virologic efficacy is defined as two negative results of SARS‐CoV‐2 in nasopharyngeal swab using RT-PCR assay with samples obtained 24 hours apart. Disease progression is defined as the need for admission to the intensive care unit, the need for mechanical ventilation, and hospital admission of previously mild cases. Adverse effect is defined as any adverse effect (side effect) reported in a study except QTc prolongation. QTc prolongation is defined as an increase of greater than 60 ms from baseline, and absolute QTc increases to greater than 500 ms.

Open Meta [Analyst] was used to analyse the proportions of mortality, QTc prolongation, and tolerability in single-arm studies [11, 12, 16, 26–29]. The pooled proportion of the outcomes was reported with its 95% confidence interval (CI). RevMan 5.3 was used to estimate the risk of virologic efficacy, mortality, disease progression, and adverse effects in studies that compared HCQ with usual care or HCQ with HCQ plus AZ. The odds ratios (ORs) and 95% CI were calculated to estimate the effect sizes. Meta‐analysis using the Mantel Hazel method was conducted, and either the fixed-effect or random-effects model was applied. A fixed‐effect model was used when the heterogeneity was low to moderate [23]; otherwise, the random‐effects model was applied.

## 5. Results

**Figure 1:**
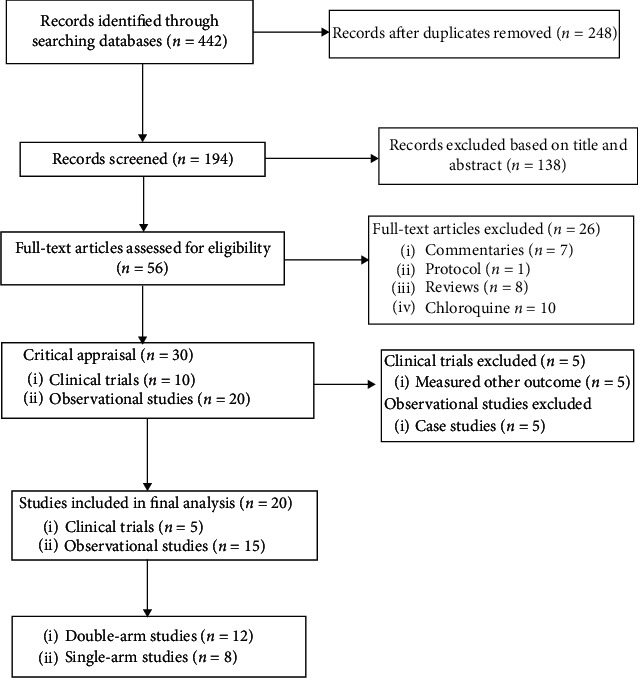
The Preferred Reporting Items for Systematic review and Meta‐Analysis (PRISMA) flow chart for the included studies.

The databases (9 databases) search produced 442 articles. After removing the duplicates and excluding 138 articles with thorough evaluation for inclusion using titles and abstracts, 56 full-text articles were assessed for eligibility. Furthermore, 30 full-text articles were subjected to critical appraisal, and 10 articles were dropped with reasons. Twenty full-text articles (5 controlled clinical trials with 288 patients and 15 observational studies with 6,742 patients) were included in the final analysis. Of these, 12 [4, 8, 13, 14, 20–22, 26, 30–32] were double-arm studies (Figure 1). These studies compared hydroxychloroquine (HCQ) either with usual/standard care or HCQ with HCQ and azithromycin (AZ). The details of the studies are described in Table 1. Two of the controlled clinical trials [20, 22] were at the preprint stage.

Both the double-arm (Table 1) and single-arm (Table 2) studies were subjected to meta-analysis to estimate the effects of the interventions. The outcomes assessed with double-arm studies include virologic efficacy [8, 20, 21], clinical efficacy (mortality [4, 8, 13, 14, 30, 31] and disease progression [4, 8, 13, 14, 22, 31, 32]), safety (risk of adverse effects) [8, 20–22], and tolerability and QT prolongation [14, 26, 34]. Outcomes were assessed for both HCQ versus standard care and HCQ versus HCQ + AZ. The outcome assessed in single-arm studies include the proportions of mortality, QTc prolongation [11, 12, 16, 26–28, 34], and drug discontinuation [12, 26–28].

### 5.1. Virologic Efficacy

**Figure 2:**
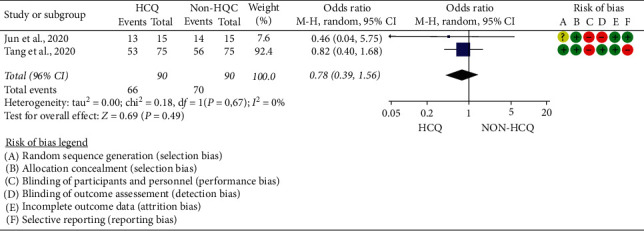
Virologic efficacy of hydroxychloroquine (HCQ) as compared to non-HCQ for patients with COVID-19.

To estimate the risk of virologic cure, the data of 180 patients (90 HCQ and 90 non-HCQ groups) from two controlled clinical trials [20, 21], with moderate risk of bias, were pooled. Although Gautret et al. [8] reported improved virologic cure rate among the HCQ group as compared to the non-HCQ group (16/20 versus 2/16), the finding was not included in the pooled data due to its serious risk of bias. Hence, the pooled result indicated that the virologic cure rate of the HCQ group was not statistically different from the non-HCQ/standard care group (OR = 0.78; 95% CI: 0.39–1.56). The test for the overall effect was *Z* = 0.69 (*p*=0.49) (Tau^2^ = 0.00; Chi^2^ = 0.18, d*f* = 1 (*p*=0.67); *I*^2^ = 0%) (Figure 2).

### 5.2. Mortality

**Figure 3:**
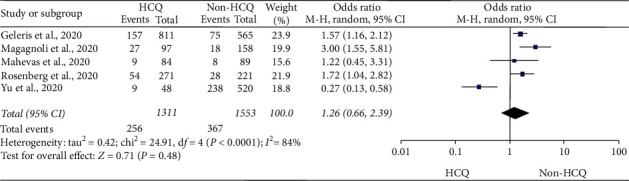
Risk of mortality among COVID-19 patients exposed to HCQ as compared to non-HCQ (standard care).

The finding was generated from five observational studies [4, 13, 14, 30, 31] comprising the data of 2,864 COVID-19 patients (1,311 HCQ and 1,553 non-HCQ groups). The overall result indicated that treatment with HCQ did not result in improved survival (OR = 1.26; 95% CI: 0.66–2.39) as compared to the routine care. The test for the overall effect, *Z* = 0.71 (*p*=0.48). However, the interpretation of this finding might be limited by the substantial heterogeneity; heterogeneity: Tau^2^ = 0.42; Chi^2^ = 24.91, d*f* = 4 (*p* ≤ 0.001); and *I*^2^ = 84% (Figure 3). A controlled clinical trial [8] also reported one death out of 14 HCQ-exposed patients and no death out of 16 in the opposite arm. Of note, the study was removed from pooled analysis as it carries a serious risk of bias (Appendix A).

**Figure 4:**
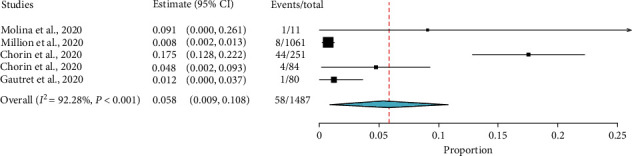
The pooled prevalence of mortality among COVD-19 patients treated with HCQ with or without AZ.

Likewise, the data of 1,487 COVID-19 patients from single-arm observational studies [12, 13, 16, 17] were included to determine the pooled prevalence of mortality among patients treated with HCQ with or without AZ. The pooled prevalence was 5.8% (95% CI: 0.9%–10.8%) with considerable heterogeneity (*I*^2^ = 92.28%, *p* < 0.001) (Figure 4). The heterogeneity could be attributed to the age difference of the COVID-19 patients, as the studies with death reports had a median age of greater than 60 years.

In the three [11, 16, 27] of the observational studies, the cause of death was respiratory and multiorgan failure. There was no death due to arrhythmogenic adverse effects.

### 5.3. Disease Progression

**Figure 5:**
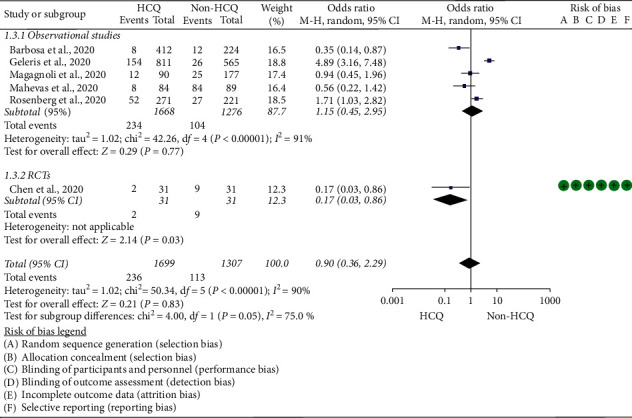
Risk of disease progression among COVID-19 patients exposed to HCQ versus non-HCQ.

The outcome coded as “disease progression” included the need for admission to the intensive care unit, the need for mechanical ventilation, and hospital admission of previously mild cases. The data of 3,003 COVID-19 patients (1,699 HCQ and 1,304 non-HCQ group) extracted from one controlled clinical trial [22] and five observational studies [4, 13, 14, 31, 32] were pooled. The overall random effect analysis indicated HCQ therapy did not appear to halt disease progression (OR = 0.9; 95% CI: 0.36–2.29). The test result for the overall effect, *Z* = 0.21 (*p*=0.83). This finding was also replicated by subgroup analysis of observational studies (OR = 1.15; 95% CI: 0.45–2.95). However, in subgroup analysis, the finding from a single controlled clinical trial (OR = 0.17; 95% CI: 0.04–0.86) was in favor of the HCQ group (Figure 5). Though the study had no risk of bias, the small sample size (31 in each arm) used made the interpretation of the finding extremely tricky. Several studies also indicated HCQ may not improve the rate of disease progression in COVID-19 patients. In a controlled clinical trial [8], which was not included in the pooled analysis, 3 patients were progressed to severe disease in the HCQ group (3/14 versus 0/14). Moreover, in three noncomparative studies [7, 17, 21, 22], out of 1,192 COVID-19 patients treated with HCQ with or without AZ, 42 patients were transferred to ICU and intubated.

### 5.4. Adverse Effects

**Figure 6:**
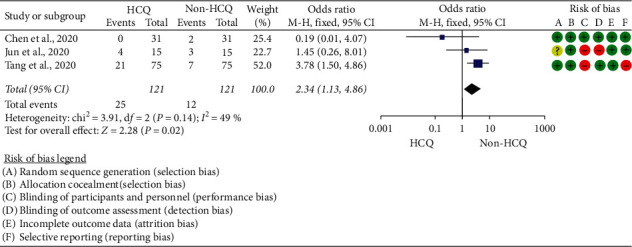
Safety of HCQ as compared to non-HCQ (standard care) among COVID-19 patients.

The data from four controlled clinical trials [8, 20–22] of 278 COVID-19 patients (141 HCQ and 137 from the non-HCQ group) were included to assess overall adverse effects (except QTc prolongation) among HCQ-exposed patients. Three controlled clinical trials [20–22] were pooled, and one controlled clinical trial [8] was described narratively due to its risk of bias A. In the pooled analysis, the odds of adverse effects among COVID-19 patients treated with HCQ patients were increased by 2.35 (OR = 2.35; 95% CI: 1.15–4.8). The test for the overall effect was statistically significant (*p*=0.02), and the summary effect of the meta-analysis was heterogeneity: Chi^2^ = 3.91, d*f* = 3 (*p*=0.27); *I*^2^ = 23% (Figure 6).

Besides, Gautret et al. [8] found more adverse effects among patients randomized to HCQ (1/14 versus 0/16).

### 5.5. QTc Prolongation

QTc prolongation was reported in two ways in most of the studies. The cut-off points, an increase in greater than 60 ms from baseline, and absolute QTc increase to greater than 500 ms were used as a threshold to discontinue medications responsible or suspected to cause QTc prolongation. These cut-off points were described by recent guidelines and FDA [18, 35]. An absolute QTc prolongation greater than 500 ms was reported in two observation studies [26, 31], which compared HCQ alone with HCQ plus AZ. A study by Rosenberg et al. [14] reported that after ECG screening, 80 patients (12.6%) had a QTc prolongation in the combination group, whereas 39 patients (16.7%) developed QTc prolongation in the HCQ group.

In the current review, the data of 291 patients from two observational studies [26, 31] were pooled to estimate the risk of QTc prolongation in patients exposed to HCQ versus HCQ plus AZ.

**Figure 7:**
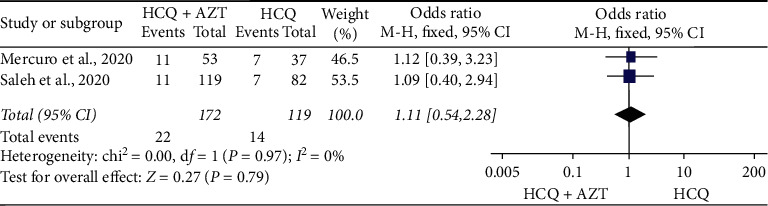
Risk of QT prolongation among COVID-19 treated with HCQ + AZ versus HCQ.

The result indicated the risk of QTc prolongation above 500 ms due to HCQ was statistically not different from those receiving HCQ plus AZ (OR = 1.11; 95% CI: 0.54–2.28) with the test for the overall effect of *Z* = 0.27 (*p*=0.79); heterogeneity: Chi^2^ = 0.00, df = 1 (*p*=0.97); *I*^2^ = 0% (Figure 7).

**Figure 8:**
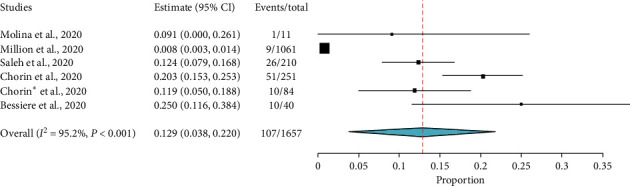
The proportion of COVD-19 patients with QTc increase by more than 60 ms from baseline after treatment with HCQ with or without AZ.

However, the findings from noncomparative studies appeared much concerning. Seven single-arm studies reported data on QT prolongation after HCQ ± AZ exposure. Six studies (1,657 patients) were reported a baseline increase in QTc by more than 60 ms [11, 12, 16, 26–28] after HCQ ± AZ exposure. The result of the analysis showed about 13.0% (95% CI: 3.8%–22%) of patients had an increase of QTc by more than 60 ms from the baseline. Considerable heterogeneity was present between studies (*Q* = 104.16, *I*^2^ = 95.2%, *p* < 0.001) (Figure 8). Several studies also raised concerns regarding QTc prolongation following HCQ exposure with or without other medications. In four studies [16, 26–28], concomitant use of other QT-prolonging medications was reported (Table 2). Million et al. [11] and Mehta et al. [36] have excluded other drugs suspected to cause QTc prolongation at baseline. Molina et al. [12] did not address the details.

**Figure 9:**
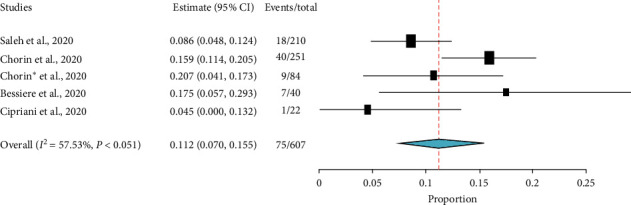
The proportion of COVD-19 patients with absolute QTc increase by more than 500 ms after treatment with HCQ with or without AZ.

A study by Mehta et al. [36] reported 4 among 22 patients had developed QTc greater than 480 ms after initiation of therapy. Similarly, from five single-arm studies [16, 26–28, 36], which comprised of 607 COVID-19 patients treated with HCQ ± AZ, about 11.2% (95% CI: 7.0%–15.5%) of patients had developed an absolute increase of QTc greater than 500 ms (Figure 9). However, a study by Million et al. [11] reported there were no patients who had an increased QTc greater than 500 ms.

A meta-regression was conducted to assess the effect of baseline use of other drugs suspected to cause QTc prolongation to above 500 ms. Indeed, the result did not show any significant effect (coefficients (Q) = −0.0; 95% CI: −0.002–0.000; *p*=0.79).

**Figure 10:**
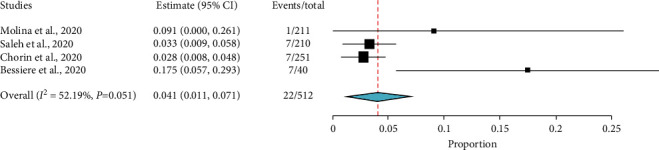
Proportion of patients' drug discontinuation due to an increase of QTC prolongation (>500 ms) in COVID-19 patients treated with HCQ with or without AZ.

In turn, the pooled prevalence of drug discontinuation due to the increased QTc prolongation to greater than 500 ms in three studies [26–28] and to greater than 60 ms from baseline in one study [12] among COVID-19 patients treated with HCQ with or without AZ was 4.1% (95% CI: 1.1–7.1). The heterogeneity among the included studies was also acceptable enough (*I*^2^ = 52.19, *p*=0.099) (Figure 10). In one study [11], three patients had discontinued the treatment due to adverse events other than QTc prolongation.

### 5.6. Admission to the Intensive Care Unit (ICU) or Death

**Figure 11:**
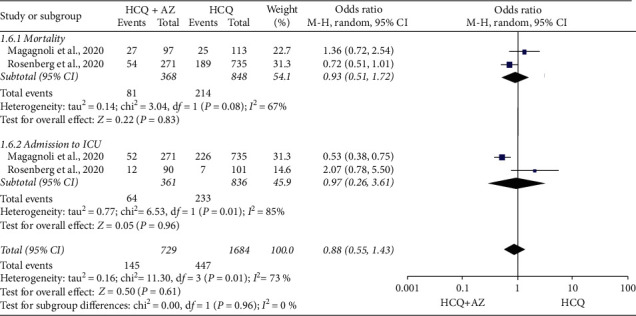
Risk of death or ICU admission in patients exposed to HCQ versus HCQ + AZ.

The data of 2,413 (729 HCQ and 1684 HCQ + AZ) COVID-19 patients were included to estimate the independent effect of AZ on mortality and ICU admission [14, 31]. Accordingly, HCQ plus AZ did not improve the risk of mortality (OR = 0.93; 95% CI: 0.51–1.72) and ICU admission (OR = 0.97; 95% CI: 0.26–3.61). The overall pooled result showed a statistically insignificant result for the composite outcome (OR = 0.88; 95% CI: 0.55–1.43). The test for the overall effect was *Z* = 0.50 (*p* = 0.61), and the heterogeneity was Tau^2^ = 0.16; Chi^2^ = 11.3, d*f* = 3 (*p*=0.01); *I*^2^ = 73% (Figure 11).

## 6. Discussion

The global community is in the state of urgency to mitigate the health and economic crisis instigated by COVID-19. Chloroquine (CQ) and its derivative hydroxychloroquine (HCQ) have traditionally been used for the treatment of malaria and certain autoimmune diseases. The drugs have possible activity against SARS-CoV-1 and SARS-CoV-2 in vitro and in clinical practice [36]. However, clinical studies were reporting contradictory results on the efficacy and safety of HCQ when used for treating COVID-19 patients. Thus, systematic review and meta-analysis of the existing studies was performed to explore the efficacy, safety, and tolerability of HCQ with or without AZ in COVID-19 patients.

The finding of this meta-analysis suggested the use of HCQ did not result in a rapid viral clearance. It also failed to improve survival and rate of disease progression. The pooled prevalence of mortality was higher in patients exposed to HCQ with or without AZ. Moreover, HCQ exposure carried a significant risk of adverse effects and a sizable proportion of patients ended up with drug discontinuation.

On the other hand, a combination of HCQ and AZ did not result in increased risk of QTc prolongation, improved survival, or preventing admission to ICU. However, the pooled proportion of observational studies indicated an alarming rate of QTc prolongation among patients receiving HCQ with or without AZ.

The finding of this study showed an absence of rapid viral clearance (OR = 0.78; 95% CI: 0.39–1.56) in patients treated with HCQ compared to the standard care. However, the review by Yang et al. [35] found better virologic efficacy with statistically insignificant results. The finding of this review was based on the data from a single study [8], which had a high risk of bias. More importantly, the former review compared HCQ + AZ with the standard care (control group). This arm of the study had a virologic cure rate of 100% reported from 6 patients. Nevertheless, the current review considered the arm which compared HCQ alone versus the control group, which reported a virologic cure rate of 57% in the HCQ arm versus 12.5% in the control arm. The study by Shamshirian et al. [37] had also questioned the virologic potency of HCQ + AZ (RR = 2.15, 95% CI: 0.31–14.77). Moreover, the optimal duration for virologic clearance is not well known. Gautret et al. [8], Jun et al. [21], and Tang et al. [20] reported a virologic cure after 6, 7, and 28 days of treatment, respectively. Therefore, drawing a conclusion from such findings irrespective of the incurred heterogeneity may be erroneous. Of note, all the included studies were open-labeled, non-randomized, and the authors did not describe the type of treatment given for the control group.

Similarly, HCQ could not demonstrate improved survival (OR = 1.26; 95% CI: 0.66–2.39) among COVID-19 patients. Gautret et al. [8] also reported an episode of death in the HCQ group, but not in the control group. Previous reviews [37–39] reported either increased mortality among HCQ groups or no statistically significant difference. As compared to our review, earlier reviews included a limited number of studies, while Sarma et al. [15] reported a combined result of mortality and clinical worsening. Our finding was not different from former studies [15, 39]. This review also found a 5.8% pooled prevalence of mortality from five observational studies [11, 12, 16, 27, 29] among COVID-19 patients treated with HCQ with or without AZ. In three [11, 16, 27] of these studies, respiratory and multiorgan failure was the cause of death. There was no reported death due to adverse effects. In all of the included studies, patients had active comorbidity. There was significant heterogeneity among the studies (*I*^2^ = 92.28%, *p* ≤ 0.001), which could be attributed to the differences in disease severity and age of the participants. Yet, meta-regression analysis did not produce any evidence.

Furthermore, HCQ therapy was not significantly associated with slowing the composite end point of disease progression (OR = 0.9; 95% CI: 0.36–2.29), unlike the finding by Chen et al. [22] (OR = 0.17; 95% CI: 0.04–0.86). The finding by Chen et al. [22] may not be dependable as it included limited number of patients (31 in each arm). The subgroup analysis result of observational studies also indicated HCQ therapy was not significantly affecting disease progression (OR = 1.15; 95% CI: 0.45–2.95). Analogous results were reported by the preceding studies [15, 38, 40]. However, one review indicated that HCQ may prevent progression to severe disease among COVID-19 patients [35]. Since the previous review included a limited number of studies, its finding must be interpreted cautiously. Our finding was in agreement with reviews by *Sarma* et al. [15] and Shamshirian et al. [37]. Nonetheless, these studies reported better outcomes of radiological progression in the HCQ arm, a finding generated from two poorly designed controlled trials [21, 22].

The safety of HCQ with or without AZ has been questioned by several studies [37, 41]. Diarrhoea, vomiting, blurred vision, rash, and headache were commonly reported adverse effects. In this review, the risk of adverse effects among COVID-19 patients treated with HCQ was 2.35 (OR = 2.35; 95% CI: 1.15–4.8). Shamshirian et al. [37] also found an increase in the odds of the adverse effect among patients exposed to HCQ (OR = 3.55, 95% CI: 1.61–7.82). Similar findings were also reported by other studies [40–42].

However, one study reported a conflicting finding where HCQ may be safe and effective, though the authors hinted more data are required for a definitive conclusion [15]. A study conducted in China reported 37.8% of ADRs in a cluster of 217 COVID-19 patients. The predominant adverse effects were drug-induced gastrointestinal disorders and liver system disorders [43]. Cardiac side effects including conduction disturbances (bundle-branch block, incomplete or complete atrioventricular block, QT prolongation, and subsequent torsade de pointes) and cardiomyopathy (hypertrophy and congestive heart failure) were also described [44, 45].

In this review, although the risk of QTc prolongation above 500 ms among those exposed to HCQ (OR = 1.11; 95% CI: 0.54–2.28) may not appear concerning, HCQ/AZ combination could be more worrisome. A finding generated from five single-arm studies [16, 26–28, 36], which comprised the data of 607 COVID-19 patients treated with HCQ ± AZ, showed 11.2% (95% CI: 7.0%–15.5%) in the pooled prevalence of QTc prolongation above 500 ms. In addition, a finding generated from other observational studies [11, 12, 16, 26–28] indicated 13.0% (95% CI: 3.8%–22%) of patients had an increase in QTc by more than 60 ms from the baseline. This finding was not similar to the data synthesized from double-arm studies. This is because of the fact that the double-arm section had compared the data of HCQ alone with HCQ + AZ. As adverse cardiovascular sequels, such as myocarditis, acute myocardial infarction, and heart failure, have been reported in COVID-19 patients and these off-label therapies are not familiar to cardiovascular clinicians managing these patients [46], emergency care physicians should outweigh the risk and the benefits. In a nutshell, numerous studies [14, 16, 17, 26, 27, 46] have raised concerns over the cardiac safety of this combination.

Drug discontinuation due to adverse effects was also a common finding. The current study showed 4.1% of patients discontinued treatment due to an increase in QTc prolongation. Another study also indicated 4.5% of COVID-19 patients treated with HCQ with or without AZ have discontinued treatment due to adverse effects. Similarly, one patient out of 117 discontinued HCQ in patients receiving HCQ with or without AZ after three days due to QTc prolongation [47].

Though the combination of HCQ + AZ was effective in some studies [15, 35], the other study [37] found it carries more hazard of death (RR = 3.65; 95% CI: 1.10–12.10) as compared to the control group. Our findings showed a lack of evidence for curbing mortality and intensive care unit admission of this combination (OR = 0.88; 95% CI: 0.55–1.45).

It is irrefutable that our study has several limitations. The inclusion of studies with a high risk of bias and methodological flaws, combining findings from controlled and uncontrolled studies, may limit its generalizability. We also reported more than one outcome, for instance, ICU admission and need of hospitalization under a single heading, namely, disease progression. This may not show the true picture of the real-world. The existence of heterogeneity, uniform treatment of all cases (mild to severe), and inclusion of limited studies with a small sample size for outcomes such as virologic efficacy may result in biased findings. We could not retrieve some of the important findings such as disease severity scale for each study and the treatments used in the standard care setting.

## 7. Conclusion

This systematic review, which included a limited number of poorly designed controlled clinical trials and several real-world studies of patients with COVID-19 requiring hospitalization, found that the use of a regimen containing HCQ with or without AZ did not offer clinical benefit. HCQ with or without AZ did not improve the rate of virologic cure, disease progression, and mortality. These regimes were associated with more adverse effects. Therefore, these drug regimens should only be used in a clinical trial setting, and a large pool of data from randomized clinical trials is warranted to have concrete evidence for safety, efficacy, and tolerability [48].

## Figures and Tables

**Figure 12:**
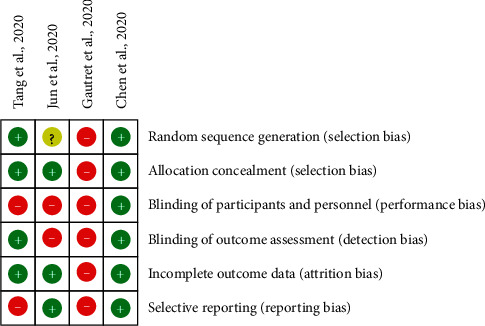
Risk of bias assessment for controlled clinical trials.

**Figure 13:**
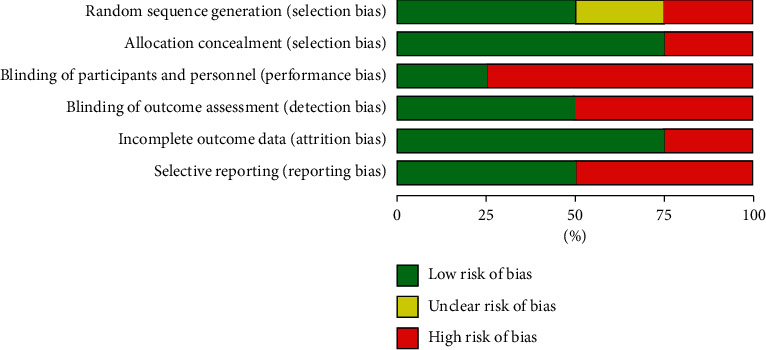
Risk of bias graph: review authors' judgments about each risk of bias item presented as percentages across all included studies.

**Table 1:** Characteristics of the studies included in the meta-analysis

Author	Study design	Population	Intervention group and protocol	Control	Outcome
Gautret et al., 2020 [8]	Open-label nonrandomized clinical trial	Age > 12	HCQ: 20 Protocol: 200 mg tid/10 days	Non-HCQ: 16	Viral cure on day 6 (14/20 vs. 2/16): HCQ group 8/14 and HCQ + AZ 6/6. Mortality: 1/14 vs. none; ICU admission: 3/14 vs. none. Adverse effects: 1/14 (nausea) vs. none
Barbosa Espe et al., 2020 [32]		Age > 18	HCQ: 412 Protocol: 800 mg/on day 1, 400 mg/6 days and AZ 500 mg/day/5 days	Non-HCQ: 224	Need for hospitalization: HCQ + AZ, 8/412 vs. 12/224
Tang et al., 2020 [20]		Age ≥ 18	HCQ group: 75 Protocol: 1.2 gd LD/d/3 days, and then, 800 mg/d for 2-3 weeks	Non-HCQ: 75	Viral cure on day 28: 53/75 vs. 56/75; adverse effect: 21/75 vs. 7/80
Jun et al., 2020 [21]		Age ≥ 18	HCQ: 15 Protocol: HCQ 400 mg qod 5/days	Non-HCQ: 15	Viral cure after day 7: 13 vs. 14; adverse effects: 4/15 vs. 3/15
Chen et al., 2020 [22]		Age ≥ 18	Non-HCQ: 15 Protocol: 400 mg/d/5 days	Non-HCQ group: 31	Clinical deterioration: 2 vs. 9; progression to severe illness: 0 vs. 4
Geleris et al., 2020 [4]	Prospective observational study	Adults	HCQ: 811 protocol: 600 mg bid day 1 and then 400 mg daily for a median of 5 days)	Non-HCQ: 562	Mortality: HCQ, 157/811; non-HCQ, 75/565 Intubated: 154/811; non-HCQ, 26/565
Rosenberg et al., 2020 [14]	Retrospective cohort	All patients	HCQ + AZ: 735 HCQ: 271 Protocol: not clear!	Non-HCQ + AZ: 221	Mortality: HCQ + AZ: 189/735; HCQ: 54/271; none: 28/221 ICU admission: HCQ: 52, non-HCQ: 27; HCQ + AZ: 226/735 QTc prolongation: HCQ + AZ: 81; HCQ: 39; none: 15 Arrhythmia: HCQ + AZ: 150; HCQ:44; and none: 23 Cardiac arrest: HCQ + AZ:114; HCQ:37; none:13
Mercuro et al., 2020 [33]	Retrospective cohort		HCQ + AZ: 53	Non-HCQ alone: 37	QTc prolongation: HCQ + AZ: 11/53 and HCQ: 7/37
Saleh et al., 2020 [26]	Prospective study		HCQ + AZ: 119	Non-HCQ: 191	QTc prolongation: HCQ + AZ: 11/119 and HCQ: 7/191
Mahévas et al., 2020 [13]	Comparative observational study	Age ≥ 18 years	HCQ: 84 Protocol: 600 mg within 48 hours of admission	Non-HCQ: 89	Mortality: 9/84 vs. 8/89. ICU admission: 8/84 vs. 14/89
Yu et al., 2020 [30]	Retrospective observational	Not specified	HCQ: 48 Protocol:200 mg bid/7–10 days	Non-HCQ: 520	Mortality: 9/48 vs. 238/520
Magagnoli et al., 2020 [31]	Retrospective observational	Not specified	HCQ: 97 HCQ + AZ: 113	Non-HCQ: 158	Mortality: HCQ: 27; HCQ + AZ: 25; no HCQ: 18. ICU admission/need for ventilation: HCQ: 12/90; HCQ + AZ: 7/101; no HCQ: 25/177

**Table 2:** Characteristics of the studies included in the meta-proportion.

Authors	Study design	Population	Intervention	Outcomes	Adverse effects of HCQ	Remark
Molina et al., 2020 [12]	Prospective, nonrandomized, noncomparative open-labeled study	(i) 11 patients (ii) Mean age 58.7 years	HCQ 600 mg/day for 10 days and + AZ 500 mg on day 1 and then 250 mg on days 2–5	After 5 days, (i) One died (ii) Two transferred to the ICU	1 patient discontinued drugs after 4 days due to QT interval prolongation from 405 ms before treatment to 460 and 470 ms under the combination	(i) Very small sample size (ii) Risk factors for QTc prolongation were not addressed (iii) The cause of death was not stated (iv) The severity of the disease was not stated

Million et al., 2020 [11]	Retrospective report	(i) 1061 patients: mean age 43.6 years (14–95 years) (ii) The majority (95.0%) of patients had a low NEWS score	HCQ 200 mg TID for 10 days HCQ+ AZ (500 mg on day 1 followed by 250 mg daily for the next 4 days) for at least 3 days	(i) Trans to ICU: 10 (ii) Death: 8 (due to respiratory failure)	(i) 25 reported mild adverse events. 3 patients discontinued treatment (due to abdominal pain, urticaria, erythematous, and bullous rash) (ii) 9 patients had a QTc prolongation of more than 60 ms from baseline but no patient exceeded 500 ms. No rhythmic, cardiac events or sudden deaths. None showing torsade de pointe	Other drugs suspected to affect QT were systematically stopped

Saleh et al., 2020 [26]	Prospective observational study	(i) 210 patients (ii) Mean age: 58.5 ± 9.1yrs (iii) Baseline QTc: 439.5 ± 24.8	HCQ: 191 (95.0%) patients (i) HCQ 400 mg PO BID for one day followed by 200 mg PO BID for 4 days ± AZ 500 mg PO or IV daily for 5 days to 119 (59.2%) patients	—	(i) TDPs due to ↑QTc = 0 patients (ii) 18 patients: ↑QTc (≥500 ms) (iii) Arrhythmogenic death = 0 patients (iv) 7 (3.5%) patients discontinued HCQ ± AZ due to ↑QTc	(i) 8 patients (4.0%) had a baseline QTc> 500 ms (ii) Receiving other QT-prolonging medications (81/210patients)

Chorin et al., 2020 [27]	Multicenter retrospective study	251 patients median age: 64 ± 13; baseline QTc (ms): 439 ± 29	(i) HCQ 400 mg BID for one day followed by 200 mg BID for 4 days HCQ+ AZ was given orally at a dose of 500 mg daily for 5 days	44 (17.5%) died of respiratory or multiorgan failure	(i) 1 TdP developed after extreme QTc prolongation (ii) 40 (15.9%) patients' extreme QTc prolongation (>500 ms) (iii) Change from baseline by > 60 ms, occurred in 51 (20.3%) patients. (iv) 7 extreme QTcpts discontinued a drug	QTc-prolonging medications (78 patients)

Chorin et al. 2020 [16]	Retrospective study	(i) 84 patients. Mean: 63 ± 15 years. (ii) Baseline average of QTc: 435 ± 24 ms	(i) HCQ 400 mg BID on the first day, followed by 200 mg BID for 5 days HCQ+ AZ 500 mg per day for 5 days	(i) 4 patients died from multiorgan failure, without evidence of arrhythmia and severe QTc prolongation (ii) 64 remained in hospital at the end (iii) 16 discharged	(i) 9 (11%) patients QTc prolonged to >500 ms (ii) 10 (12%) patients had increased >60 ms (iii) No tdp events	Receiving other suspected QTc prolonging drugs, 32 (39%) patients

Bessière et al. 2020 [28]	Retrospective study	(i) 40 critically ill patients (ii) median (IQR), 68 (58–74) yrs (ii) Baseline QTc, median (IQR), ms 414 (392–428) Excluded: QTc greater than 460 milliseconds	(i) HCQ 200 mg, bid/10 days) ± AZ 250 mg/d for 5 days (ii) HCQ alone 22 (55%) patients (iii) HCQ and AZ 18 (45%) patients	After treatment initiation, 30 (75%) patients required invasive mechanical ventilation	(i) After 2–5 days QTc ≥500 ms or ΔQTc>60 ms (*n* = 14 patients) (ii) No ventricular arrhythmia, including torsade de pointes The antiviral treatment ceased before completion for 7 patients (17.5%) following ECG abnormalities	(i) Lack of generalizability beyond the ICU (ii) Use of other QTc prolonging drugs (propofol, amiodarone, ciprofloxacin, and ondansetron), 20 (50%) patients

Gautret et al. 2020 [8]	Prospective observational study	(i) 80 patients (ii) Median: 52.5 (18–88) years (iii) 92% low NEWS score (iv) QTc prolonging medical conditions and drugs excluded at baseline	HCQ 200 mg of PO, TID for 10 days. HCQ + azithromycin 500 mg on D1 followed by 250 mg per day for the next 4 days	Transfer to ICU: 3 (3.8%), where 2 were improved and 1 returned to IDW. Death: 1 (1.2%). Discharged/improved: 65 (81.2%); currently hospitalized: 14 (1 in ICU and 13 in IDW)	(i) Possible adverse events: 7 (8.7%) (ii) Nausea or vomiting: 2 (2.5%) (iii) Diarrhea 4 (5.0%) Blurred vision: 1 (1.2%)	(i) Patients followed-up for at least six days were included in analysis (ii) Max. 10 days Lost to follow-up patients were not known

Cipiriani et al. 2020 [34]	Observational case-control study	(i) 22 patients (ii) Median age: 64 (iii) Baseline QTc-interval: 426 (403–447) ms Controls: 34 health individuals, matched for age and sex	HCQ 200 mg BID and AZ 500 mg, once daily for at least three days	—	(i) QTc prolongation (QTc ≥ 480 ms) after HCQ treatment: 4 (18%) patients, of which 1 patient developed > 500 ms (ii) No cases of syncope, fatal arrhythmias, and sudden cardiac death	(i) Conditions predisposing to QTc prolongation including medications were excluded (ii) There was a significant QTc difference after HCQ initiation (426 vs. 450 ms, *p* = 0.02) (iii) Mean of QTc of patients was 453 (439–477) ms while of controls was 407 (397–418); *P* value <0.001.

HCQ, hydroxychloroquine; AZ, azithromycin; CQ, chloroquine; IDW, infectious disease ward; NEWS, National Early Warning Score; ↑, increase; PO, oral; BID, twice daily; ms, milliseconds. Discontinuation of a subject from a clinical trial should be considered if there is an increase in QT/QTc to >500 ms or if >60 ms over baseline are commonly used as thresholds for potential discontinuation. Guidance for Industry E14 Clinical Evaluation of QT/QTc Interval Prolongation and Proarrhythmic Potential for Non-Antiarrhythmic Drugs.

**Table 3:** Modified Newcastle-Ottawa Quality Assessment Scale for the included observational studies.

Selection^a^					Comparability^b^	Outcome^c^			
Included studies	Representativeness of the exposed cohort	Selection of nonexposed	Ascertainment of exposure	Outcome of interest was not present at start of study		Assessment of outcome	Length of follow-up	Adequacy of follow-up	Total number of stars^d^
Rosenberg et al., 2020 [14]	A^*∗*^	A^*∗*^	A^*∗*^	A^*∗*^	A^*∗*^B^*∗*^	A^*∗*^	B	A^*∗*^	8

Mahévas et al., 2020 [13]	A^*∗*^	A^*∗*^	A^*∗*^	A^*∗*^	A^*∗*^B^*∗*^	A^*∗*^	B	B^*∗*^	8

Barbosa Espe et al., 2020 [32]	A^*∗*^	A^*∗*^	A^*∗*^	A^*∗*^	B^*∗*^	A^*∗*^	B	B^*∗*^	7

Geleris et al., 2020 [4]	A^*∗*^	A^*∗*^	A^*∗*^	A^*∗*^	A^*∗*^B^*∗*^	A^*∗*^	B	B^*∗*^	8

Saleh et al., 2020 [26]	A^*∗*^	A^*∗*^	A^*∗*^	A^*∗*^	B^*∗*^	A^*∗*^	B	D	6

Mercuro et al., 2020 [33]	B^*∗*^	A^*∗*^	A^*∗*^	A^*∗*^	B^*∗*^	A^*∗*^	B	C	6

Magagnoli et al., 2020 [31]	B^*∗*^	A^*∗*^	A^*∗*^	A^*∗*^	A^*∗*^B^*∗*^	A^*∗*^	B	D	7

Yu et al., 2020 [30]	A^*∗*^	A^*∗*^	A^*∗*^	A^*∗*^	B^*∗*^	B^*∗*^	B	D	6

Mehta et al., 2020 [36]	A^*∗*^	A^*∗*^	A^*∗*^	A^*∗*^	A^*∗*^B^*∗*^	A^*∗*^	A^*∗*^	A^*∗*^	9

^a^Selection: (1) representativeness of the exposed cohort: A, consecutive eligible participants were selected, participants were randomly selected, or all participants were invited to participate from the source population; B, not satisfying requirements in part (a) or not stated. (2) Selection of the nonexposed cohort: A, selected from the same source population;*∗* B, selected from a different source population; C, no description. (3) Ascertainment of exposure: A, structured injury data (e.g., record completed by medical staff); *∗*B, structured interview; *∗*C, written self-report; D, no description. (4) For a demonstration that the outcome of interest was not present at the start of the study: *∗*A, yes; B, no or not explicitly stated. ^b^Comparibility: for comparability of cohorts based on the design or analysis: *∗*A, study controls for previous injury; *∗*B, study controls for age. ^c^Outcome: (1) assessment of outcome: A, independent or blind assessment stated or confirmation of the outcome by reference to secure records (e.g., imaging and structured injury data); *∗*B, record linkage (e.g., identified through ICD codes on database records); *∗*C, self-report with no reference to original structured injury data or imaging; D, no description. (2) Was follow-up long enough for outcomes to occur? *∗*A, yes (≥3 months); B, no (<3 months). (3) Adequacy of follow-up of cohorts: A*∗*, complete follow-up—all participants accounted for; *∗*B, subjects lost to follow-up unlikely to introduce bias (<15% lost to follow-up, or description provided of those lost*∗*); C, follow-up rate <85% and no description of those lost provided; D, no statement. ^d^Total is out of 9 stars. Note: ≥7, high-quality study; 5–7, moderate quality study; <5, low-quality study.

## Data Availability

Additional data not included in the article can be obtained from the corresponding author upon request.

## Appendix

### A. Search Strategy

The search strategy was created with the assistance of the librarians at the Jimma University drug information center. The Mendeley desktop was used as a reference manager.

#### 1. PubMed

((((2019-ncov) OR (2019 Novel corona virus)) OR (COVID-19)) OR (Coronavirus-19)) AND (((Hydroxychloroquine) OR (Plaquenil)) OR (Hydroxychloroquine sulphate))

1. 2019-ncov
2. 2019 Novel corona virus
3. COVID-19
4. Coronavirus-19
5. Hydroxychloroquine
6. Plaquenil
7. Hydroxychloroquine sulphate [*MeSH*]
8. 1 OR 2 OR 3 OR 4
9. 6 OR 7 OR 8
10. 8 AND 9…70 articles

#### 2. Cochrane CENTRAL

((((2019-ncov) OR (2019 Novel corona virus)) OR (COVID-19)) OR (Coronavirus-19)) AND (((Hydroxychloroquine) OR (Plaquenil)) OR (Hydroxychloroquine sulphate))

1. 2019-ncov
2. 2019 Novel corona virus
3. COVID-19
4. Coronavirus-19
5. Hydroxychloroquine
6. Plaquenil
7. Hydroxychloroquine sulphate
8. 1 OR 2 OR 3 OR 4
9. 6 OR 7 OR 8
10. 8 AND 9

#### 3. Web of Science

((((2019-ncov) OR (2019 Novel corona virus)) OR (COVID-19)) OR (Coronavirus-19)) AND (((Hydroxychloroquine) OR (Plaquenil)) OR (Hydroxychloroquine sulphate))

1. 2019-ncov
2. 2019 Novel corona virus
3. COVID-19
4. Coronavirus-19
5. Hydroxychloroquine
6. Plaquenil
7. Hydroxychloroquine sulphate
8. 1 OR 2 OR 3 OR 4
9. 6 OR 7 OR 8
10. 8 AND 9

#### 4. EMBASE

((((2019-ncov) OR (2019 Novel corona virus)) OR (COVID-19)) OR (Coronavirus-19)) AND (((Hydroxychloroquine) OR (Plaquenil)) OR (Hydroxychloroquine sulphate))

1. 2019-ncov
2. 2019 Novel corona virus
3. COVID-19
4. Coronavirus-19
5. Hydroxychloroquine
6. Plaquenil
7. Hydroxychloroquine sulphate
8. 1 OR 2 OR 3 OR 4
9. 6 OR 7 OR 8
10. 8 AND 9

### (B) Risk of Bias Assessment Result for Included Studies

Randomized controlled trials and the Modified Newcastle-Ottawa Quality Assessment Scale for the included observational studies (Figures 12 and 13 and Table 3).
